# Prevalence and phenotype of diabetes and prediabetes using fasting glucose vs HbA1c in a Caribbean population

**DOI:** 10.7189/jogh.07.020407

**Published:** 2017-12

**Authors:** Nigel Unwin, Christina Howitt, Angela MC Rose, T Alafia Samuels, Anselm JM Hennis, Ian R Hambleton

**Affiliations:** 1 George Alleyne Chronic Disease Research Centre, Caribbean Institute for Health Research, The University of the West Indies, Bridgetown, Barbados; 2 MRC Epidemiology Unit, University of Cambridge, Cambridge, United Kingdom

## Abstract

**Background:**

Both fasting plasma glucose (FPG) and HbA1c are recommended for the diagnosis of diabetes and prediabetes by the American Diabetes Association (ADA), and for diabetes by the World Health Organization. The ADA guidance is influential on clinical practice in many developing countries, including in the Caribbean and Latin America. We aimed to compare the prevalence and characteristics of individuals identified as having diabetes and prediabetes by FPG and HbA1c in a predominantly African ancestry Caribbean population.

**Methods:**

A representative population–based sample of 1234 adults (≥25 years of age) resident in Barbados was recruited. Standard methods with appropriate quality control were used to collect data on height, weight, blood pressure, fasting lipids and history of diagnosed diabetes, and to measure fasting glucose and HbA1c. Those with previously diagnosed diabetes (n = 192) were excluded from the analyses. Diabetes was defined as: FPG ≥7.0 mmol/L or HbA1c ≥6.5%; prediabetes as: FPG ≥5.6 to <7mmol/L or HbA1c ≥5.7 to <6.5%.

**Results:**

Complete data were available on 939 participants without previously diagnosed diabetes. The prevalence of undiagnosed diabetes was higher, but not significantly so, by HbA1c (4.9%, 95% CI 3.5, 6.8) vs FPG (3.5%, 2.4, 5.1). Overall 79 individuals had diabetes by either measure, but only 21 on both. The prevalence of prediabetes was higher by HbA1c compared to FPG: 41.7% (37.9, 45.6) vs 15.0% (12.8, 17.5). Overall 558 individuals had prediabetes by either measure, but only 107 on both. HbA1c, but not FPG, was significantly higher in women than men; and FPG, but not HbA1c, was significantly associated with raised triglycerides and low HDL cholesterol.

**Conclusion:**

The agreement between FPG and HbA1c defined hyperglycaemia is poor. In addition, there are some differences in the phenotype of those identified, and HbA1c gives a much higher prevalence of prediabetes. The routine use of HbA1c for screening and diagnosis in this population would have major implications for clinical and public health policies and resources. Given the lack of robust evidence, particularly for prediabetes, on whether intervention in the individuals identified would improve outcomes, this approach to screening and diagnosis cannot be currently recommended for this population.

Globally there are estimated to be over 400 million people with diabetes, and of these 4 out of 5 live in low and middle income countries [1]. Diabetes is estimated to cause 5 million deaths per year, and is a major contributor to premature adult mortality [1]. A substantial proportion of people with Type 2 diabetes, which comprises approximately 90–95% of all persons with diabetes, are undiagnosed. This undiagnosed proportion can be as high as 80–90% in some of the poorest settings, such as in many of the countries of sub–Saharan Africa, and is typically around 20–30% in the world’s richest countries, including most of those in Western Europe and North America [1].

In addition to individuals with diabetes, there are those with ‘prediabetes’ [2], also known as ‘intermediate hyperglycaemia’ [3]. These individuals have glucose levels that are above normal, but below the diagnostic threshold for diabetes. They are at increased risk of developing diabetes. There are three broad categories of prediabetes, and there is excellent evidence that among persons with one category (impaired glucose tolerance, which is based on the 2–hour post challenge result in an oral glucose tolerance test) the risk of developing diabetes can be substantially lowered through changes in diet and physical activity or the use of metformin [4].

There have been two major changes over the past 20 years to the diagnostic criteria for diabetes. In 1997 the American Diabetes Association (ADA) lowered the diagnostic cut–point for fasting plasma glucose from 7.8 mmol/L to 7 mmol/L, and recommended that fasting glucose becomes the main diagnostic test, rather than the more time–consuming and expensive oral glucose tolerance test, which includes collection of a fasting sample and a sample 2 hours after a 75g glucose load [5]. In 2009, the International Expert Committee, which included the ADA, recommended that the diagnosis could also be made using glycated haemoglobin, HbA1c, at a cut–point of 6.5% [6]. HbA1c reflects the exposure of red cells to glucose over their lifetime. In a person with normal haemoglobin the average lifespan of red cells is around 120 days [7], with the average age of red cells in circulation being in the order of 40–60 days [8]. HbA1c therefore can be interpreted as reflecting average glucose levels over this time. The major advantage of HbA1c is that it does not require the person to be fasting, but it has the disadvantage of being more expensive to measure, and that the result can be influenced by medical conditions that affect red cell turnover and/or produce abnormal haemoglobin [9]. The World Health Organization (WHO) followed in lowering the cut–point for fasting glucose and in accepting HbA1c as a diagnostic test in 1999 [10] and 2011 [9], respectively. However, unlike the ADA, WHO still emphasizes the desirability of continuing to use an oral glucose tolerance test as the gold standard for the diagnosis of diabetes, while acknowledging that the use of fasting glucose or HbA1c is more practical.

While the ADA and WHO diagnostic criteria for diabetes are identical, there are two major differences when it comes to the diagnosis of prediabetes (the ADA term) or intermediate hyperglycaemia (the WHO term). First, the ADA cut–point for prediabetes based on fasting plasma glucose (FPG) at 5.6 mmol/L [11] is lower than that recommended by WHO at 6.1 mmol/L [3]. Second, WHO does not believe that there is enough evidence to recommend using HbA1c to diagnose prediabetes [9], whereas the ADA does and defines it as an HbA1c level from 5.7 to <6.5% [11]. It is relevant to the aims of the work present here to note that the ADA recommends that all people with prediabetes, whatever the method of diagnosis, are entered into a structured diabetes prevention programme [11]. Thus, if changing the approach to diagnosis changes the numbers of individuals identified, this would have implications for health care resources.

The Caribbean is an ethnically and linguistically diverse region [12], that includes 18 English–speaking countries and territories that are part of the Caribbean Community (CARICOM) [13] and have some of the highest rates of diabetes and diabetes mortality in the Americas [1,14]. Barbados, where the work described here was undertaken, is part of the English–speaking Caribbean and a member of CARICOM. It is an independent country, with a population of around 280 000, over 90% of whom are of African ancestry [15]. While the diagnosis of diabetes in Barbados and other parts of the Caribbean to date has largely relied on fasting glucose, ADA guidance [11] is highly respected and influential, and the greater availability of accessible and good quality HbA1c assays means that it is increasingly being used.

In this paper we make use of a recently conducted population–based survey [16] in which all participants had both FPG and HbA1c measured. Our aim was to assess what difference, if any, would using HbA1c rather than FPG make to the identification of diabetes and prediabetes. Specifically, we sought to determine whether there would be a difference in prevalence of the conditions, the individuals identified and the characteristics of those individuals. Our aim was not to determine which measure better predicts complications or progression (that would require a very different type of study), but rather to determine what the local policy and resource implications might be of using one approach compared to the other. This, as far as we can determine, is the first study of its type conducted in the Caribbean.

## METHODS

### Overview of the study design and data collection

The Health of the Nation survey was a cross–sectional study of the Barbadian population aged 25 years and older, which aimed to provide estimates of the prevalence and social distribution of non–communicable diseases (NCDs) and their risk factors. Data were collected for 1234 participants on health conditions and behaviours, as well as sociodemographic information. Data collection took place between September 2011 and May 2013. The survey methods have been described in detail elsewhere [16] and are summarised here.

On behalf of the Ministry of Health of the Government of Barbados, and in collaboration with the Barbados Statistical Service, a multistage sampling process was used, with enumeration districts (EDs) selected with a probability proportional to their population size, households randomly selected within each ED, and a single eligible participant randomly selected from each household using the Kish method [17]. Staff from the Barbados Statistical Service made the first contact with each household and, if permission was given, the household details were passed on to members of the Health of the Nation survey team.

Data collection took place in participants’ homes across two visits. At the first visit a standard questionnaire was completed by interview. Items collected included whether the participant had been told by a doctor that they had diabetes, and if so what type of treatment they were on. At this first visit, height, weight, waist and hip circumferences were recorded, and blood pressure was measured using an Omron HEM–705CP digital machine. Standard protocols were followed for all measurements, and data collection staff were trained and accredited in their use.

At the end of this first visit a second early morning visit was arranged for the collection of a venous blood sample, typically on the day after the first visit. The participant was instructed to fast overnight for at least 9 hours, drinking nothing other than water. The venous sample was collected into two tubes: a sodium fluoride tube and an EDTA tube. The sodium fluoride tube was placed immediately on wet ice, and the EDTA tube was placed in a cool box, which contained ice packs.

### Assays of glucose, HbA1c and lipids

The sodium fluoride tube was kept on wet ice until its contents were analysed for plasma glucose later that morning. This analysis was undertaken at the Barbados Reference Laboratory using the glucose hexokinase method on Roche Cobas 6000 (Roche Diagnostics, Mannheim, Germany).

The sample in the EDTA tube was used for HbA1c and lipid analyses. All analyses were conducted during the same morning as the sample collection. Lipids (total cholesterol, HDL cholesterol and triglycerides) were analysed using a Reflotron biochemical analyser (Roche Diagnostics, Mannheim, Germany). HbA1c was analysed using a DCA 2000 analyser (Siemens Health Care Diagnostics, Munich, Germany), a method certified by NGSP and directly traceable to the DCCT reference [18] and which is not affected by common haemoglobin variants such as HbC and HbS [19]. Manufacturers’ recommended quality control procedures for all biochemical assays were followed throughout.

The Reflotron and DCA 2000 analyser were used as they provide assay methods that are practical, affordable, widely used in surveys and known to be of good quality compared to laboratory standard methods [20–23]. However, we wished to be able to align the HbA1c and lipid results with those from the Barbados Reference Laboratory, an internationally accredited laboratory (with the American College of Pathologists – CAP). This was achieved by analysing duplicate samples, 56 for HbA1c and 50 for lipids. For HbA1c, this was done at the time of venepuncture by collecting blood into two separate EDTA tubes, with one tube going to the reference laboratory, where HbA1c was measured using high performance liquid chromatography (HPLC). For the lipids, 50 plasma aliquots, which had been stored at minus 80°C, were assayed in the reference laboratory for total, HDL and LDL cholesterol and triglycerides on a Roche Cobas 6000 analyzer.

Details of the comparison between the values of HbA1c and the lipids assessed by the two different assay methods are given in the supplementary material. There were small but statistically significant differences between the results from the survey assay methods and those in the Barbados Reference Laboratory. As would be expected, there was inter–individual variation in the size of the differences. These are expressed as the standard deviation of the differences [24] and show that 95% of the differences for the lipids lay within 0.2 and 0.7 mmol/L (Table S1 in **Online Supplementary Document(Online Supplementary Document)**) of the mean, and for HbA1c within 1% (Table S1 in **Online Supplementary Document(Online Supplementary Document)**). As described in the supplement, simple linear regression equations were applied to the survey results to align them with reference laboratory values.

### Statistical analyses

Data were analysed using Stata statistical software (version 13, StataCorp, College Station, Texas). Weights were applied to account for the sampling design, non–response at the ED level, and to match the age and sex distribution of the Barbadian population according to the 2010 census [15]. As described elsewhere, compared with the official population, provided by the 2010 Barbados Population and Housing Census, the survey generally under–sampled young adults and oversampled the elderly, and more women than men took part. These discrepancies were addressed by the weighting scheme, full details of which are available on–line in the publication by Howitt et al [16].

Categories of hyperglycaemia were defined according to the criteria of the ADA [11] and the WHO [3,9]. Diabetes was defined, according to both ADA and WHO criteria, as FPG ≥7.0 mmol/L or HbA1c ≥6.5%. Prediabetes according to ADA criteria was defined as FPG ≥5.6 to <7 mmol/L or HbA1c ≥5.7 to <6.5%. Prediabetes (impaired fasting glucose) according to WHO criteria was defined as FPG of ≥6.1 to <7 mmol/L.

Those with previously diagnosed diabetes (n = 192) were excluded from the analyses in which the classification and characteristics according to FPG and HbA1c values were compared. Agreement was investigated in unweighted analyses in which categories based on FPG and HbA1c were cross–tabulated. Unweighted kappa statistics, using the Kappa command in Stata, were calculated.

The characteristics of individuals with ADA–defined hyperglycaemia were investigated as follows. First, differences (with 95% confidence intervals) in means and proportions between individuals with hyperglycaemia (ie, above the prediabetes cut–points) and normoglycaemia were examined. This was performed separately for the HbA1c and FPG defined categories. Age and sex were compared, plus anthropometric and biological variables commonly associated with hyperglycaemia [25]: body mass index, waist circumference, systolic and diastolic blood pressure, HDL cholesterol and triglycerides. The association of normo/hyperglycaemia with hypertension, as a binary variable (yes/no), was also examined, in order to allow for the fact that some individuals may have normal blood pressure readings because they are on treatment. Hypertension was defined as being on [or using prescribed] medication for hypertension and/or systolic blood pressure >140 mm Hg and/or diastolic blood pressure >90 mm Hg. Second, logistic regression was undertaken to identify which characteristics were independently associated with hyperglycaemia. A backward step selection process was used, starting with all the independent variables in the model and removing the least significantly associated variable at each step. This was halted when all variables in the model were associated with a *P* value of at least <0.05. All the analyses were undertaken using the survey module in Stata, in order to apply appropriate weights given the sampling design, non–response and age and sex distribution of the Barbados population (as described above). Model specification was evaluated using the link test and goodness of fit using the ‘estat gof’ command [26].

### Ethical considerations

Written informed consent was obtained from all participants. The study was approved by the Research Ethics Committee of the University of the West Indies, Cave Hill and the Barbados Ministry of Health. All persons in the study found to have abnormal results, including abnormal HbA1c or FPG, were informed and advised to seek medical advice and further investigation as needed.

## RESULTS

There was a total of 1234 respondents (470 men and 764 women). Out of 2277 eligible households contacted by the Barbados Statistical Service, 1646 (72.3%) agreed to having their details passed on to the Health of the Nation survey; from these, 1234 (75.0%) individuals participated in the study (see Howitt et al. for full details [16]).

Diagnosed diabetes was reported by 192 participants, 11.4% (95% confidence interval (CI) 8.3, 15.5) of men and 15.8% (95% CI 13.1, 18.8) of women (**Table 1**). Of the 1042 without a previous diagnosis of diabetes, complete data on fasting glucose and HbA1c were available on 939 (90%). In these individuals, the prevalence of previously undiagnosed diabetes ranges from 3.5% (95% CI 2.4, 5.1) (FPG criterion in men and women) to 7.3% (95% CI 5.0, 10.4) (FPG and/or HbA1c criterion in men and women). The prevalence of previously undiagnosed diabetes is higher by the HbA1c criterion compared to the FPG in both women and men; and higher in women compared to men by both the HbA1c and FPG criteria. However, the confidence intervals are wide and overlapping.

**Table 1:** Prevalence of categories of diabetes and prediabetes in Barbadian adults (25 years and over)*

	Men (n = 430)	Women (n = 701)	All (n = 1131)
**Diagnosed diabetes (n = 192):**			
On hypoglycaemic medication	9.2 (6.6, 12.8)	13.8 (11.4, 16.7)	11.7 (9.8, 13.8)
Not on medication	2.1 (1.1, 4.1)	1.9 (1.0, 3.7)	2.0 (1.2, 3.3)
All	11.4 (8.3, 15.5)	15.8 (13.1, 18.8)	13.8 (11.7, 16.2)
**Undiagnosed diabetes:†**			
FPG ≥7.0 mmol/L	3.3 (2.0, 5.5)	3.7 (2.2, 6.3)	3.5 (2.4, 5.1)
HBA1c ≥6.5%	3.9 (2.3, 6.5)	5.7 (3.9, 8.3)	4.9 (3.5, 6.8)
On either of above	5.7 (3.9, 8.3)	7.3 (5.0, 10.4)	6.5 (4.9, 8.6)
**Prediabetes (WHO criteria):**			
FPG ≥6.1 to <7 mmol/L	5.5 (3.5, 8.5)	4.4 (2.8, 6.7)	4.9 (3.5,6.9)
**Prediabetes (ADA criteria):†**			
FPG ≥5.6 to <7 mmol/L	16.7 (12.9, 21.2)	13.6 (10.6, 17.2)	15.0 (12.8, 17.5)
HBA1c ≥5.7 to <6.5%	39.7 (33.8, 46.0)	43.4 (39.8, 47.2)	41.7 (37.9, 45.6)
On either of above	42.2 (35.9, 48.8)	45.8 (42.2, 49.4)	44.1 (40.5, 47.9)

FPG – fasting plasma glucose, HBA1c – haemoglobin A1c, ADA – American Diabetes Association

*Figures are percentages (95% confidence intervals), and are weighted for sampling design, non–response and the age structure of the Barbados adult population.

†See **Table 2** for numbers of individuals (unweighted) by category.

The prevalence of prediabetes differs markedly by the three different sets of criteria (**Table 1**). The lowest prevalence is for the WHO FPG criterion, with an overall prevalence in men and women of 4.9% (95% CI 3.5, 6.9). Applying the ADA FPG criterion triples the prevalence, to 15.0% (95% CI 12.8, 17.5). Applying the ADA HbA1c criterion gives more than double the ADA FPG prevalence, at 41.7% (95% CI 37.9, 45.6). Defining prediabetes based on either FPG or HbA1c gives a prevalence of 44.1% (95% CI 40.5, 47.9).

The agreement between diabetes and prediabetes according to ADA FPG vs HbA1c criteria is poor, reflected by low kappa values (**Table 2**). For example, there are 43 individuals with previously undiagnosed diabetes based on FPG, and 57 based on HbA1c, but only 21 on both (kappa 0.39, 95% CI 0.32, 0.45). With prediabetes there are 170 based on FPG, 495 on the HbA1c, and only 107 on both (kappa 0.14, 95% CI 0.10, 0.19).

**Table 2:** Agreement between categories of diabetes and prediabetes based on fasting plasma glucose (FPG) and HbA1c criteria*

	FPG			
**HbA1c:**	**Not Diabetes**	**Diabetes**	**Total**	
Not diabetes	860	22	882	
Diabetes	36	21	57	
Total	896	43	939	
Kappa (95% CI)	0.39 (0.32, 0.45)			
	**ADA Categories based on FPG**			
**ADA categories based on HbA1c**	**Normal**	**Prediabetes**	**Diabetes**	**Total**
Normal	342	37	8	387
Prediabetes	374	107	14	495
Diabetes	10	26	21	57
Total	726	170	43	939
Kappa (95% CI)	0.14 (0.10, 0.19)			

CI – confidence interval, FPG – fasting plasma glucose, HBA1c – haemoglobin A1c, ADA – American Diabetes Association

*Figures are numbers of study participants, and kappa statistics (95% confidence intervals).

Anthropometric and biological characteristics of those without a previous diagnosis of diabetes are summarised in **Table 3**. There are notable differences between men and women, with women having a significantly (based on the lack of overlap between 95% confidence intervals) higher body mass index, HbA1c, total and HDL cholesterol, but similar fasting glucose, and lower triglycerides and lower mean systolic blood pressure. A higher proportion of women than men are on treatment for hypertension. The prevalence of obesity (BMI ≥30 kg/m^2^) is 43.5% (95% CI 39.1, 48.0) in women and 23.2% (95% CI 18.4, 28.8) in men.

**Table 3:** Characteristics of those without previously diagnosed diabetes, and with complete fasting glucose and HbA1c data*

	Men	Women	All
**Age in years (n, column %):**			
25–44	139 (38.5)	252 (43.6)	391 (41.6)
45–64	155 (42.9)	237 (41.0)	392 (41.8)
65+	67 (18.6)	89 (15.4)	156 (16.6)
All	361 (100.0)	578 (100.0)	939 (100.0)
**Glucose & HbA1c:**			
Mean fasting glucose (mmol/L)	5.2 (5.1, 5.3)	5.2 (5.1, 5.4)	5.2 (5.2, 5.3)
Mean HbA1c (%)	5.7 (5.6, 5.7)	5.8 (5.7, 5.9)	5.7 (5.7, 5.8)
**Other:**			
Anthropometry			
Mean BMI (kg/m^2^)	26.4 (25.7, 27.1)	29.8 (29.1, 30.5)	28.2 (27.8, 28.6)
Mean waist circumference (cm)	90.1 (88.1, 92.0)	92.6 (91.2, 94.1)	92.1 (91.1, 93.1)
**Blood pressure:**			
Mean sBP (mmHg)	131.0 (129.1, 133.0)	126.5 (124.5, 128.6)	128.7 (127.3, 130.1)
Mean dBP (mmHg)	77.8 (76.3, 79.3)	76.8 (75.7, 77.8)	77.3 (76.4, 78.2)
% On treatment for hypertension	14.1 (10.9, 18.1)	25.3 (20.4, 30.8)	19.9 (16.7, 23.6)
**Lipids:**			
Mean total cholesterol (mmol/L)	4.6 (4.5, 4.7)	4.9 (4.8, 4.9)	4.7 (4.7, 4.8)
Mean HDL cholesterol(mmol/L)	1.28 (1.25, 1.31)	1.40 (1.38, 1.43)	1.34 (1.32, 1.36)
Mean triglycerides (mmol/L)	0.96 (0.92, 1.00)	0.88 (0.85, 0.92)	0.92 (0.90, 0.94)

HBA1c – haemoglobin A1c, BMI – body mass index, sBP – systolic blood pressure, dBP – diastolic blood pressure, HDL – high density lipoprotein

*103 individuals of the 1042 without previously diagnosed diabetes had missing fasting glucose and/or HbA1c values. All figures, with the exception of the numbers by age group, are weighted for the sampling design, non–response, and the age structure of the Barbados adult population.

Selected characteristics of those with ADA–defined hyperglycaemia compared to those with normoglycaemia by FPG and HbA1c criteria are shown in **Table 4**. Older age, higher body mass index, waist circumference and blood pressure are all related to both FPG and HbA1c categories of hyperglycaemia. Higher triglyceride, and lower HDL cholesterol levels are associated with FPG, but not HbA1c, defined hyperglycaemia. Female sex is associated with HbA1c, but not FPG, defined hyperglycaemia.

**Table 4:** Comparison of selected characteristics between groups with normal and raised fasting glucose or HbA1c based on ADA criteria*

	Normal	Raised	Absolute difference (95% CI)
**Fasting glucose:**			
Age (years)	45.2 (0.8)	53.7 (1.5)	8.5 (5.5, 11.4)
Female sex (%)	52.7%	50.1%	–2.8 (–12.3, 6.7)
BMI (kg/m^2^)	27.5 (0.4)	30.1 (0.6)	2.5 (1.0, 4.0)
Waist (cm)	89.8 (0.8)	97.0 (1.2)	7.0 (3.9, 10.2)
sBP (mmHg)	126.6 (0.7)	135.9 (1.9)	9.2 (5.3, 13.0)
dBP (mmHg)	76.4 (0.5)	80.3 (1.1)	4.1 (1.8, 6.3)
Hypertension (%)†	28.4	56.8	28.3 (19.0, 37.6)
HDL cholesterol (mmol/L)	1.36 (0.01)	1.29 (0.02)	–0.07 (–0.12, –0.02)
Triglycerides (mmol/L)	0.97 (0.02)	1.17 (0.06)	0.20 (0.07, 0.34)
**HbA1c:**			
Age (years)	41.3 (0.8)	51.9 (0.9)	10.9 (8.6, 13.2)
Female sex (%)	46.8%	56.6%	9.6 (2.5, 16.6)
BMI (kg/m^2^)	26.5 (0.4)	29.5 (0.4)	3.0 (1.7, 4.2)
Waist (cm)	87.0 (1.0)	94.9 (0.8)	7.7 (4.9, 10.5)
sBP (mmHg)	124.8 (1.0)	131.8 (0.9)	7.4 (4.8, 10.1)
dBP (mmHg)	75.9 (0.8)	78.4 (0.6)	2.8 (1.0, 4.7)
Hypertension (%)†	22.2	44.9	22.7 (15.3, 30.0)
HDL cholesterol (mmol/L)	1.34 (0.02)	1.34 (0.02)	0.00 (–0.05, 00.1)
Triglycerides (mmol/L)	0.96 (0.03)	1.05 (0.03)	0.09 (0.00, 00.2)

HBA1c – haemoglobin A1c, BMI – body mass index, sBP – systolic blood pressure, dBP – diastolic blood pressure, HDL – high density lipoprotein

*Figures are means (standard error) unless otherwise indicated.

†On BP lowering medication and/or blood pressure ≥140/90. See text for details.

Backward step logistic regression, as described in the methods section and starting with all the variables shown in **Table 4** was undertaken to identify factors independently associated with FPG and HbA1c categories of hyperglycaemia. The FPG category was independently associated with age, waist circumference, hypertension and triglycerides (**Table 5**). The HbA1c category was associated with age, waist circumference and female sex, but not blood pressure, HDL cholesterol or triglycerides. In further analysis, waist circumference was entered into the model for HbA1c as a binary variable using sex specific cut–points (94 cm for women, 102 cm for men [27]). As shown in **Table 5**, this had the effect of reducing the association with female sex (odds ratio (OR) 1.25, 95% CI 0.94, 1.66).

**Table 5:** Results of logistic regression to identify independent associations between hyperglycaemia and the characteristics in **Table 4**

	Odds Ratio	95% CIs
**Fasting glucose:**		
Age (years)	1.03	1.01, 1.04
Waist (cm)	1.03	1.01, 1.05
Hypertension (Y/N)	2.03	1.25, 3.30
Triglycerides (mmol/L)	1.46	1.07, 2.00
**HbA1c:**		
Age (years)	1.05	1.04, 1.07
Female sex	1.40	1.03, 1.91
Waist (cm)	1.04	1.02, 1.06
**HbA1c** (model with sex specific “high waist”*):		
Age (years)	1.06	1.04, 1.07
Female sex	1.25	0.94, 1.66
High waist (Y/N)	2.32	1.50, 3.58

HBA1c – haemoglobin A1c

*≥94 cm in women; ≥102 cm in men.

## DISCUSSION

In the first study of its type in the Caribbean, we compared the prevalence and agreement of diabetes and prediabetes as defined by FPG and HbA1c. We found that HbA1c gave a higher prevalence of both diabetes (non–significant) and prediabetes. Applying the ADA criteria, the prevalence of prediabetes was more than twice as high based on HbA1c compared to fasting glucose. The agreement between the two methods of classification was poor. There were also differences in the factors associated with HbA1c and FPG–defined hyperglycaemia. HbA1c, but not FPG, defined hyperglycaemia was associated with female sex. Raised FPG, but not HbA1c, defined hyperglycaemia was associated with raised triglycerides and hypertension. At a population level, therefore, there are important differences in terms of prevalence and the characteristics of the individuals identified.

While both HbA1c and FPG are recommended by the ADA and WHO for the diagnosis of diabetes, and by the ADA for the diagnosis of prediabetes, it is unclear whether one should be preferred over the other. Analyses of cross–sectional data from nine studies across five countries found that both FPG and HbA1c at the currently recommended diagnostic cut–points for diabetes were strongly associated with an increased risk of diabetic retinopathy, whereas the 2–hour glucose from an oral glucose tolerance test was not [28]. Prediabetes, whether defined by HbA1c or FPG, is associated with an increased risk of developing diabetes [29]. However, it does not appear that one test is better than the other at predicting harder adverse outcomes, such as death or incident cardiovascular disease. For example, neither adds much to the prediction of cardiovascular events when other established cardiovascular risk factors are taken into account [30].

It is also unclear, given the currently available evidence, whether the lifestyle and pharmacological interventions that have been shown to substantially reduce the incidence of diabetes in those with impaired glucose tolerance [4], which requires an oral glucose tolerance test for diagnosis, are also effective in those with prediabetes based on FPG or HbA1c. It seems a reasonable assumption that they would be, and indeed guidance is based on this assumption [11]. However, hard evidence is lacking, and what does exist suggests that in the case of impaired fasting glucose these interventions may be either ineffective or much less effective [31,32]. Comparable studies targeting prediabetes based on HbA1c do not exist [29].

Other studies have also investigated differences in the prevalence of diabetes and prediabetes by different measures of glycaemia, whether HbA1c, FPG, or the use of an oral glucose tolerance test. A pooled analysis to investigate the impact on diabetes prevalence, based on 63 health examination surveys, found differences in prevalence (both higher and lower), with similar prevalences in only a minority (15%) [33]. Differences in agreement between studies were significantly, but weakly, related to population differences in body mass index and gross domestic product [33]. For example, in a pooled regression analysis, the prevalence of diabetes by HbA1c, while controlling for the prevalence by FPG, was positively related with age, mean body mass index, and gross domestic project [33].

Studies reporting differences in the prevalence of prediabetes by FPG and HbA1c have also found differences in both directions. In the Canadian Health Measures Survey, for example, differences in the prevalence of prediabetes in adults based on ADA criteria by FPG and HbA1c are similar to what we describe here: 13% using FPG vs 33% with HbA1c [34]. In the United States (US) NHANES study, by contrast, the prevalences of prediabetes were almost the reverse: 28.7% with FPG and 12.4% with HbA1c [35]. The reasons for these differences are not clear based on the published data. Neither study presented a comparison by sex, nor compared the characteristics of those with differently defined prediabetes. The higher prevalence of obesity in the US (30–33%) compared to Canada (20–22%) [36] is not obviously related to these differences. Interestingly, in a relatively small study (n = 216) of predominantly male (mean age 37 years) African migrants to the US, the prevalence of hyperglycaemia (taking the figures from **Table 2** in the paper) [37] using ADA criteria was 13% by FPG and 35% by HbA1c, similar to what we report in our study here.

It is also relevant to our findings to note that some analyses, in particular based on data from the US NHANES study, have described higher levels of HbA1c in black compared to white participants (‘black’ and ‘white’ being the terms used in the paper) independent of differences in fasting and post challenge glucose [38]. Similarly, in the Atherosclerosis Risk in Communities Study, HbA1c levels were higher in black compared to white participants independent of fasting glucose level [39]. The American Diabetes Prevention Program (conducted in adults with impaired glucose tolerance) also reported differences by ethnicity in average HbA1c levels, with higher levels in black compared to white participants, and these were independent of differences in obesity, fasting and post challenge glucose, and insulin resistance [40]. It may be, therefore, that relationships between measures of glucose (including fasting and post challenge) and HbA1c differ by ethnic group, with a higher HbA1c level in people of black African origin compared to people of white European origin [41,42]. The reasons for such differences remain unclear and include potential racial/ethnic differences in susceptibility to glycation of haemoglobin [41,42], and underlying average glucose concentrations being higher in African descent populations (and not properly accounted for by adjusting for fasting and post challenge glucose).

One little mentioned but possible contributory factor to population differences in HbA1c levels is iron deficiency. This may increase red cell life and thus HbA1c levels [43,44]. It is possible that iron deficiency anaemia in women of child–bearing age contributes to the higher HbA1c levels in women found in our study. Unfortunately, haemoglobin was not measured in the survey, but WHO estimates for the prevalence of iron deficiency anaemia in Barbados suggest that around one in five to one in four women aged 15–49 are anaemic [45]. The potential effect of iron deficiency anaemia on HbA1c levels requires further evaluation. Another, possibly more important, contributory factor to higher HbA1c levels in women compared to men in Barbados is the higher prevalence of obesity, particularly abdominal obesity. Controlling for abdominal obesity, defined using sex–specific cut–points, reduced to non–significant the association of HbA1c defined hyperglycaemia with female sex.

Before drawing conclusions on the implications of the findings reported here, we should acknowledge both the strengths and limitations of the study. A strength is that this is a representative population–based study. Standardised methods were used to collect all data, most importantly to measure FPG and HbA1c. The method of HbA1c measurement used is approved by the NGSP [18] and widely used, and in addition we aligned these values to those from the only reference laboratory in Barbados that uses the gold standard approach of HPLC. Limitations of the study include that it was a relatively small study population, and thus lacks precision to properly investigate some areas of interest, including differences in diabetes prevalence by FPG or HbA1c. In addition, it was an epidemiological study using epidemiological definitions of prediabetes and undiagnosed diabetes; ie, our definitions are based on a single measurement, whereas in clinical practice, and in the absence of clear symptoms, a confirmatory diagnostic test should always be performed [3,11]. Finally, it is worth noting that the kappa statistic to assess agreement between categories based on FPG and HbA1c requires careful interpretation. Kappa values are influenced by the prevalence of the different categories, and when the prevalence is very different (such as the prevalence of diabetes vs no diabetes), Kappa may provide an overly negative summary of the agreement [46]. We recommend therefore that the Kappa values are only used and interpreted alongside the actual cross–tabulated data (**Table 2**). Despite these limitations, our findings demonstrate marked differences between FPG and HbA1c in the identification of hyperglycaemia in the adult population of Barbados.

In conclusion, current guidance recommends the use of both FPG and HbA1c for the diagnosis of diabetes and prediabetes. This study has shown that according to ADA guidance around 44% of the adult population of Barbados has prediabetes, with a further 6.5% having previously undiagnosed diabetes. When previously diagnosed diabetes is added, roughly two out of three (64%) adults are classified as having either diabetes or prediabetes and thus the majority of the adult population would be recommended for clinical intervention [11]. By contrast, if WHO diagnostic guidance is followed then roughly one in four adults (25%) has either diabetes or prediabetes. In very well–resourced settings it might be considered prudent to measure both FPG and HbA1c and manage individuals accordingly, as is recommended [11]. However, in less well–resourced settings, such as in Barbados and most of the Caribbean, our findings indicate that the increasing use of HbA1c to diagnose diabetes and prediabetes could have major implications: for human and financial resources; clinical practice and public health policies; and for health systems that are already struggling to cope with the high and increasing burden of chronic diseases. In addition, there is limited evidence to support whether one approach is better than the other for identifying individuals who will benefit from treatment. In short, the currently available guidance is particularly unhelpful, and potentially damaging (if it leads to less effective use of scarce resources), for situations in which resources are limited. We propose therefore that a pragmatic approach, guided by the currently available evidence, is to use FPG as the diagnostic test for both diabetes and prediabetes and to restrict the label of ‘prediabetes’ to the narrow range recommended by WHO. Expansion of the prediabetes category, whether by adopting the broader ADA FPG range or using HbA1c criteria, should be contingent upon clear evidence that cost–effective interventions to improve outcomes are available [29].
