# Supplementary material for: Prevalence and phenotype of diabetes and prediabetes using fasting glucose vs HbA1c in a Caribbean population
Source: J Glob Health. 2017 Sep 13;7(2):020407. doi: 10.7189/jogh.07.020407 (PMC5604098; doi:10.7189/jogh.07.020407)
Supplement: Online Supplementary Document [file jogh-07-020407-s001.pdf]

Online Supplementary Document

Unwin et al. Prevalence and phenotype of diabetes and prediabetes using fasting glucose versus HbA1c in a Caribbean population

J Glob Health 2017;7:020407

Comparison of HbA1c Measurements by the survey instrument (DCA 2000) and HPLC in the Barbados Reference Laboratory

Table S1 – Mean difference in HbA1c, 95% confidence intervals and standard deviation (SD), n=56

	r	Mean difference[a]	95% CIs on difference	SD of difference
HbA1c	0.95	0.37 %	0.24, 0.51	0.51

[a]DCA 2000 result minus HPLC result

Adjustment made to HbA1c values and rationale

Adjustment was made to provide values and prevalence estimates (i.e. prevalence of undiagnosed diabetes and of pre-diabetes) that are closer to those that would be expected if all the HbA1c measurements had been made in the Barbados Reference Laboratory. This was done by deriving a regression equation, using the Reference Laboratory value as the dependent value.

The equation that was applied is:

HbA1c = DC*0.83 + 0.081

Where DC is the value from the DCA 2000 analyser.

Clearly, this is an imperfect adjustment but provides prevalence figures that are closer to what one would expect to find had the Barbados Reference Laboratory Assay been used on all survey participants.

Comparison of the lipid measurements by the survey instrument (Reflotron) and the Roche Cobas 6000 in the Barbados Reference Laboratory

Table S2 – Correlation coefficient (r), mean difference (mmol/l), 95% confidence intervals and standard deviation for total cholesterol, HDL cholesterol and triglyceride values (n=49)

	r	Mean difference[a]	95% CIs on difference	SD of difference
Total cholesterol	0.96	-0.41 mmol/l	-0.50, -0.31	0.34
HDL cholesterol	0.84	-0.45 mmol/l	-0.51, -0.39	0.21

Triglycerides	0.98	0.12 mmol/l	0.09, 0.15	0.10

[a]Reflotron value minus Cobas 6000 value

Adjustment made to lipid values and rationale

Adjustment was made therefore to provide mean values that are closer to those that would be expected had all the measurements been undertaken in the Barbados Reference Laboratory. This was done by deriving regression equations, using the reference laboratory result as the dependent variable and the reflotron result as the independent variable. The follow equations were applied to the reflotron values (rv):

Total cholesterol = rv * 1.08 + 0.04

HDL cholesterol = rv * 0.76 + 0.66

Triglycerides = rv * 1.05 - 0.19
